# Time-driven interventions for affective disorders and resistance to treatment

**DOI:** 10.47626/2237-6089-2020-0178

**Published:** 2021-10-22

**Authors:** Marsal Sanches

**Affiliations:** 1 UT Health Center of Excellence on Mood Disorders Faillace Department of Psychiatry & Behavioral Sciences McGovern Medical School Houston TX USA UT Health Center of Excellence on Mood Disorders, Faillace Department of Psychiatry & Behavioral Sciences, McGovern Medical School, Houston, TX, USA.

The editorial titled “Time-driven interventions for affective disorders: on resonance and the Oberth effect”^1^ raises important points related to the potential role of time and biological rhythms in therapeutic strategies targeting mood disorders. Considering, on the one hand, the classic concept of mood disorders as displaying a cyclic, recurrent nature, and, on the other hand, the more recent constructs describing affective illnesses (more specifically, bipolar disorder) according to a staging model, time seems to correspond to a critical, albeit often neglected, element. Moreover, the authors utilize a curious analogy from astronautics, the Oberth effect, to illustrate the importance of taking into consideration the appropriate timing when implementing and combining specific therapeutic interventions, aiming at optimizing their efficacy.

A natural extension (or, in a certain way, the “mirror image”) of the authors’ observations would be the importance of timing in determining resistance or lack of response to certain therapeutic modalities. The concept of resistance to treatment in mood disorders is often based on dose and duration of treatment (in the case of pharmacological interventions) and on number of sessions (when it comes to psychotherapeutic interventions). These parameters are of critical importance for purposes of treatment planning and can also have research implications, as failing to respond to certain treatments is often listed as a criterion to include (or exclude) participants from clinical trials. Similarly, insurance companies commonly require documentation of lack of previous response to other treatments before approving newer and more costly interventions, such as transcranial magnetic stimulation. Using the same rationale utilized by the authors, failing to respond to cognitive behavioral therapy (CBT) should be characterized not only in terms of number of sessions but also considering the timing of the sessions. For example, a patient is unlikely to respond to CBT (or any other type of psychotherapy) if experiencing a severe depressive episode with catatonic features. Likewise, symptom seasonality in bipolar disorder is well described,^2^ and seasonal variations in mood can potentially act as confounders while analyzing treatment response in patients with mood disorder or rates of relapse during the maintenance period.

Despite the obviousness of these statements, timing is rarely mentioned during the characterization of a patient’s treatment history and response. The authors’ observations are extremely commendable and draw special attention to the importance of time-related issues at different levels during the treatment of mental illnesses, especially mood disorders. Contrarily to some positions from physics and philosophy that argue for the non-existence of time,^3^ time should be considered a crucial factor during the elaboration of a psychiatric treatment plan.

## References

[1] 1. Argolo FC, Gadelha A, Pan PM, Bressan RA. Time-driven interventions for affective disorders: on resonance and the Oberth effect. Trends Psychiatry Psychother. 2020;42:113-4. 10.1590/2237-6089-2019-0067 32520168

[2] 2. Geoffroy PA, Bellivier F, Scott J, Etain B. Seasonality and bipolar disorder: a systematic review, from admission rates to seasonality of symptoms. J Affect Disord. 2014;168:210-23. 10.1016/j.jad.2014.07.002 25063960

[3] 3. Jaffe A. Stop all the clocks. Nature. 2018;556:304-5.

